# Motivation factors for suicidal behavior and their clinical relevance in admitted psychiatric patients

**DOI:** 10.1371/journal.pone.0176565

**Published:** 2017-04-26

**Authors:** Naoki Hayashi, Miyabi Igarashi, Atsushi Imai, Yuka Yoshizawa, Kaori Asamura, Yoichi Ishikawa, Taro Tokunaga, Kayo Ishimoto, Yoshitaka Tatebayashi, Hirohiko Harima, Naoki Kumagai, Hidetoki Ishii, Yuji Okazaki

**Affiliations:** 1 Department of Psychiatry, Teikyo University School of Medicine, Tokyo, Japan; 2 Tokyo Metropolitan Chubu Comprehensive Center for Mental Health and Welfare, Tokyo, Japan; 3 Department of Psychiatry, Tokyo Metropolitan Matsuzawa Hospital, Tokyo, Japan; 4 Tokyo Metropolitan Mental Health and Welfare Center, Tokyo, Japan; 5 Kabukicho Clinic, Tokyo, Japan; 6 Affective Disorders Research Team, Tokyo Metropolitan Institute of Medical Science, Tokyo, Japan; 7 Graduate School of Education and Human Development, Nagoya University, Nagoya, Japan; 8 Michinoo Hospital, Nagasaki, Japan; Universita Cattolica del Sacro Cuore Sede di Roma, ITALY

## Abstract

**Background:**

Suicidal behavior (SB) is a major, worldwide health concern. To date there is limited understanding of the associated motivational aspects which accompany this self-initiated conduct.

**Aims:**

To develop a method for identifying motivational features associated with SB by studying admitted psychiatric patients, and to examine their clinical relevance.

**Methods:**

By performing a factor analytic study using data obtained from a patient sample exhibiting high suicidality and a variety of SB methods, Motivations for SB Scale (MSBS) was constructed to measure the features. Data included assessments of DSM-IV psychiatric and personality disorders, suicide intent, depressive symptomatology, overt aggression, recent life events (RLEs) and methods of SB, collated from structured interviews. Association of identified features with clinical variables was examined by correlation analyses and MANCOVA.

**Results:**

Factor analyses elicited a 4-factor solution composed of Interpersonal-testing (IT), Interpersonal-change (IC), Self-renunciation (SR) and Self-sustenance (SS). These factors were classified according to two distinctions, namely interpersonal vs. intra-personal directedness, and the level of assumed influence by SB or the relationship to prevailing emotions. Analyses revealed meaningful links between patient features and clinical variables. Interpersonal-motivations (IT and IC) were associated with overt aggression, low suicidality and RLE discord or conflict, while SR was associated with depression, high suicidality and RLE separation or death. Borderline personality disorder showed association with IC and SS. When self-strangulation was set as a reference SB method, self-cutting and overdose-taking were linked to IT and SS, respectively.

**Conclusions:**

The factors extracted in this study largely corresponded to factors from previous studies, implying that they may be useful in a wider clinical context. The association of these features with SB-related factors suggests that they constitute an integral part of the process leading to SB. These results provide a base for further research into clinical strategies for patient management and therapy.

## Introduction

Suicidal behavior (SB) is a worldwide, major health problem placing a great burden on medical services [1]. An investigation revealed that in Japan, approximately five percent of patients who presented for emergency treatment had intentionally caused physical damage to themselves [2]. SB is a potent indicator for the at-high risk condition of suicide and frequently necessitates intensive psychiatric treatment [1]. Another feature of SB is that it is principally, a self-initiated and deliberately performed act, with clear motivational aspects. While a number of studies have addressed the motivation, often as self-reported reasons or motives, further objective scrutiny of these motivations is needed for a better understanding and ultimately better therapies for patients.

Since suicide intent is an axial feature among the motivations, SB is in general classified by the presence or absence of intent. One extreme situation would be characterized by a suicide attempt in which suicide intent dominates the motivation. At the opposite end would be non-suicidal, self-injurious behavior (NSSI) or self-injurious behavior (SIB), in which the suicide intent is principally negated or unquestioned. Most types of SB lie between these two extremes of the spectrum.

Although suicide intent is central in the assessment of SB, there are other motivations linked to this behavior, and identifying these factors is important for deepening our understanding of SB. The classical work of Bancroft et al. [3] examined the reasons for overdose-taking among patients admitted to hospital as emergencies, and found that motivations to influence people around them or to regulate distressing inner conditions played a role in their decision making. Thereafter, the inquiry items listed by Bancroft et al. [3, 4] have been widely used in factor analytic studies for exploring the contributory factors and clinical significance of motivations [5–7]. In contrast, studies on NSSI or SIB found different results. Suyemoto [8] noted functions in SB that are hypothesized to have positive effects, and to increase the tendency of SB. Nock and Prinstein [9] analyzed self-reported data from adolescent inpatients and proposed the 4-factors of motivation model: Social (interpersonal) positive and negative reinforcements, and autonomic (intra-personal) positive and negative reinforcements. Many subsequent studies have followed this line of investigation [10–12].

Suicidal behavior is the end product of complex interactions between diverse SB-related factors such as motivation, emotion and interpersonal conditions at the time of conducting SB. From the cognitive-motivational-relational theory of emotion [13], motivation is hypothesized to be formed in parallel with other factors and to exert influences on SB. In previous studies [14, 15], negative emotions expressed in the form of aggression and depression have been seen as motivation-forming factors. The interaction of motivation with interpersonal recent life events (RLE) and SB method selection are also of clinical interest.

Additionally, some psychiatric disorders are thought to influence motivation. SB is particularly common among patients with borderline personality disorder (BPD) [16]. The motivational characteristics in BPD are defined as complex [17] and controlling emotions and influencing others [15, 18]. Likewise, depressive disorders are also related to the motivations since they frequently promote suicidal thoughts and attempts in affected individuals.

Motivations vary depending on the type of SB and the sample population. To obtain an overarching perspective on the motivational aspects of SB, establishing clinically relevant assessment methods applicable across various populations are crucial. The identification and relevance of motivation factors in SB remain poorly understood, as to date, only limited investigations have been conducted.

In this study, by analyzing data from a sample of psychiatric patients that exhibited high suicidality (i.e., prevailing suicidal intent [19] and a high suicide rate (6%) in 2 year-follow-up period [20]), a relatively low level of physical damage caused by SB and a great variety of SB methods prior to admission [19], and using a newly devised self-reporting scale for assessing the motivation, we aimed to determine the principal features of the motivation and their interrelationship with other SB-related factors, such as negative emotions, recent life events and psychiatric disorders. In addition, this study also attempted to examine the applicability of our assessment model across diverse SB-types and populations.

## Methods

### Subjects

The subjects included in this study were patients who were consecutively admitted to Tokyo Metropolitan Matsuzawa Hospital (TMMH) during a 20-month period from April 2006 to November 2007, showing SB within a 2-week period prior to admission. Participants were identified by screening intake records of admitted patients and asking physicians to complete an inquiry sheet recording whether their patients exhibited SB that met the definition of de Leo et al. [21]: “A non-habitual act with non-fatal outcome that the individual, expecting to, or taking the risk, to die or to inflict bodily harm, initiated and carried out for the purpose of bringing about wanted changes.”

The inclusion criteria were, (1) age at admission equal to or older than 20 years, (2) a hospital stay longer than three days, (3) absence of any prominent intelligence disability or organic brain damage, (4) fluency in Japanese, (5) ability to comprehend the study procedures and undergo study assessments and (6) provision of written informed consent for study participation, and in cases of involuntary admission, additional written informed consent from a family member or guardian.

### Development of Motivations for SB Scale (MSBS)

The original item set of MSBS included inquiries from previous investigations related to overdose-taking [4] and SIB [9], and items found by asking open-ended questions about the motivation in our preliminary study of 20 suicidal patients. Items with a response rate higher than 10 percent were selected for the use in this study. The original 27 item set was administered in study interviews. All the items were rated on the 3-point scale: clearly present, 2; unclear, 1; not present, 0. Test-retest reliability of items in the MSBS was examined in a subsample of 25 participants by calculating the intraclass correlation coefficient (ICC). For this reliability study, the rating was conducted twice with an interval of between 7 to 10 days.

### Assessment

1. Interview schedule of SB prior to admission and in lifetime history [19]
  Methods of SB used immediately prior to admission, together with methods, number and time points of SB in a patient’s life history were recorded. All chosen methods of SB were ascertained as individual questions in interviews. In this study, the methods of SB were classified into self-cutting, overdosing, self-strangulation and other methods. When more than one method was used in a recent SB episode, the method causing the most severe physical damage was recorded.
2. Structured Clinical Interview for DSM-IV Axis I Disorders, Clinician Version (SCID-I, CV) [22] and Structured Clinical Interview for DSM-IV Axis II Personality Disorders (SCID-II) [23]
  The presence of psychiatric and personality disorders based on the Diagnostic and Statistical Manual of Mental Disorders, Fourth Edition (DSM-IV) were determined by conducting SCID-I CV and SCID-II interviews. Presence or absence of the frequent psychiatric disorder groups and personality disorders were used in analysis.
3. Beck Depression Inventory-II (BDI) [24] and Beck Hopelessness Scale (BHS) [25]
  BDI is a 4-point, 21-item self-report scale for assessing depressive symptoms. BHS, a self-report scale for measuring hopelessness, is composed of 20 true-false items. Total scores were used in subsequent analyses.
4. Suicide Intent Scales (SIS) [26]
  SIS is a 20-item, semi-structured instrument designed to record information on a suicidal person’s wish to die at the time of a suicide attempt. In this study, a scale composed of the first 15 SIS items was used to rate the intensity of suicide intent based on the circumstances and patient’s reports of thoughts and feelings.
5. Overt Aggression Scale-Modified (OAS-M) [27]
  OAS-M is 6- or 7-point, 9-item clinician-administered, semi-structured interview designed to measure manifestations of 3 domains: aggression, irritability and suicidal tendencies of subjects. In this study, behavior within a week prior to admission was rated using this scale. In subsequent analyses, scores from the 3 domains were used. Additionally, OAS-M item 7b concerning lethality of SB was used to determine SB method classification of participants when more than one method was used in the SB episode.
6. Interview schedule of Recent Life Events (RLEs) [19]
  Items within the RLE assessment were selected from the studies of Heikkinen, et al. [28]. The RLEs were divided into 3 domains: close interpersonal relations, life situations and health conditions. Interpersonal RLEs were classified in terms of the nature of the relationship, namely: spouse or partner, other family members or other close individuals. The quality of interpersonal RLEs was also recorded in terms of presence and absence of discord or conflict, and separation or death. In this study, the quality classification of interpersonal RLEs within 3 months prior to admission was used.

All assessments, including MSBS were administered in the study interviews. Details relating to the assessments in this study are provided elsewhere [19].

### Statistical analysis

To determine the basic model of MSBS, explorative factor analysis (EFA) based on maximum likelihood (ML)-extraction with Promax oblique rotation was performed. Only items with sufficient reliability (ICC > 0.5) in the original MSBS were entered into EFA. Items with high factor loading (factor loading > 0.5) were included in the model. To ensure a simple structure, cross-loaded items were removed from the model. Subsequent to this, confirmatory factor analysis (CFA) based on ML parameter estimates was performed. In accordance with the software modification index to reduce the model’s χ2 statistic, we repeated to add an inter-error covariance path until the model produced admissible values in the 2 goodness-of-fit indices: comparative fit index (CFI) greater than 0.95, and root mean square error of approximation (RMSEA) smaller than 0.07 [29]. Cronbach’s alpha coefficient was used to assess the internal consistency of MSBS. The composite subscale scores of each factor were calculated for further analyses.

To examine convergent validity and clinical relevance of MSBS subscales, correlation analyses with SB-related variables and Multivariate Analyses of Covariance (MANCOVAs) was conducted, with variables of frequent psychiatric disorder groups, interpersonal RLEs and SB methods as factors, and gender and age (years) as covariates.

In the analysis, software packages of SPSS 16.0.2 (SPSS, 2008) and IBM AMOS 22.0.0 (IBM, SPSS, 2013) were used. We applied a significance level of 0.05, and two-tailed probability in correlation analyses.

### Ethical procedure

This study was approved by the ethical committee of TMMH on 28 March 2006.

## Results

### Description of sample

From 3450 psychiatric admissions to TMMH during the study period, 292 cases (280 patients) with SB were identified. From the 225 patients who fulfilled the inclusion criteria (1–5), 155 (68.9%) consented to participate in the study, and completed assessments.

The study comprised 68 men and 87 women. Their mean ages (SDs) were 36.4 (11.8) and 36.6 (12.1) years, respectively. Participants living alone numbered 92 (59.4%), while those living with a spouse or partner numbered 37 (23.9%). A total of 82 subjects (52.9%) were unemployed, while 125 (81.3%) attained an educational level equal to, or higher than middle high-school graduation.

Table 1 presents frequent SB methods, psychiatric and personality disorder groups and interpersonal RLEs for each subject. Participants who used the three most frequent SB methods immediately prior to admission constituted over three quarters of our study sample. The median (range) for SB episodes in a lifetime history was 7 (1–141). One hundred and eleven (71.6%) subjects had more than 3 episodes in their lifetime history. The number (%) of participants with at least one SB episode of overdosing, self-cutting and self-strangulation in their lifetime history, was 99 (63.9%), 106 (68.4%) and 37 (23.9%), respectively.

**Table 1:** Clinical characteristics of subjects.

	Male (N = 68) N (%)	Female (N = 87) N (%)	Total (N = 155) N (%)
**Methods of SB: Overdosing** ^a^ **/ Self-cutting** ^b^ **/ Self-strangulation** ^c^ **/ Others** ^d^	19 (27.9) / 26 (38.2) / 6 (8.8) / 17 (25.0)	23 (26.4) / 30 (34.5) / 15 (17.2) / 19 (21.8)	42 (27.1) / 56 (36.1) / 21 (13.5) / 36 (23.2)
**DSM-IV Mood disorders** ^e^^,^ ^i^	36 (52.9)	60 (69.0)	96 (61.9)
**DSM-IV Anxiety disorders** ^f^^,^ ^j^	28 (41.2)	58 (66.7)	86 (55.5)
**DSM-IV Substance-related disorders** ^g^^,^ ^k^	24 (35.3)	35 (40.2)	59 (38.1)
**DSM-IV BPD** ^l^	28 (41.2)	58 (66.7)	86 (55.5)
**DSM-IV AVPD** ^h^^,^ ^m^	21 (30.9)	28 (32.2)	49 (31.6)
**RLE Discord or conflict** ^n^	33 (48.5)	64 (73.6)	97 (62.6)
**RLE Separation or death**	18 (26.5)	27 (31.0)	45 (29.0)

SB: Suicidal behavior, RLE: Recent life events, BPD: Borderline personality disorder, AVPD: Avoidant personality disorder

^a^ Prescribed psychotropics; 37 (23.9%), other prescribed medicine; 3 (1.9%), OTC medicine; 6 (3.9%).

^b^ Cutting of wrist or forearm; 38 (24.5%) and other part(s) of the body; 24 (15.5%).

^c^ Hanging; 12 (7.7%) and other self-strangulation; 9 (5.8%).

^d^ Jumping from a height; 18 (11.6%), attempting death in traffic 13; (8.4%) and others; 5 (3.2%).

^e^ Major depressive disorders; 67 (43.2%), bipolar I and II disorders; 21 (13.5%).

^f^ Panic disorders; 53 (34.2%), PTSD; 25 (16.1%).

^g^ Alcohol-related disorders; 41 (26.5%) and non-alcoholic substance-related disorders; 28 (18.1%).

^h^ Other frequent types of personality disorder (PD) were antisocial PD; 42 (27.1%) and obsessive-compulsive PD; 34 (21.9%).

^i^ Male subjects were less common than females among subjects with mood disorders (p = 0.047, Exact test).

^j^ Subjects with anxiety disorders were younger than subjects without these disorders The means (SDs) (years) were 33.8 (9.8) vs. 39.8 (13.5) (F_1, 153_ = 10.235, p = 0.002, ANOVA).

^k^ Subjects with substance-related disorders were younger than subjects without these disorders (33.3 (8.5) vs. 38.5 (13.5), F_1, 153_ = 7.144, p = 0.008, ANOVA).

^l^ Male subjects were less common than females among subjects with BPD (p = 0.002, Exact test). Subjects with BPD were younger than non-sufferers (32.7 (7.7) vs. 41.3 (14.5), F_1, 153_ = 22.537, p < 0.001, ANOVA).

^m^ Subjects with AVPD were younger than non-sufferers (33.6 (8.6) vs. 37.8 (13.0), F_1, 153_ = 4.327, p = 0.039).

^n^ Male subjects were less common than females among subjects with RLE discord or conflict (p = 0.002, Exact test). Subjects with RLE discord or conflict were younger than those without this RLE (34.8 (10.8) vs. 39.1 (13.4), F_1, 153_ = 4.574, p = 0.034, ANOVA).

A psychiatric diagnosis of mood disorder or anxiety disorder was present in over half of the subjects. BPD was the most frequent PD, exhibited by over half of the subjects. One hundred and thirty five (87.1%) subjects had at least one PD type. Shown in the notes for Table 1 are the diagnoses and other clinical variables with a significant association to gender or age. Mood disorders and BPD occurred more frequently in female than in male subjects. Subjects with anxiety disorders and BPD were younger than unaffected subjects. Analyses also showed correlations between psychiatric disorders (groups). The phi coefficients between affective disorders and anxiety disorders, affective disorders and BPD and anxiety disorders and BPD were 0.234, 0.314 and 0.451, respectively (all p values < 0.003). Over 60 percent of subjects reported RLE discord or conflict. Particularly, this RLE was common among younger aged females. In addition, RLE discord or conflict, and separation or death were weakly associated with a phi coefficient of 0.171 (p = 0.033).

The means (SDs) of BDI and BHS total scores were 30.5 (12.3) and 13.2 (4.8), respectively. Severe level scores (30–63 points) of depressive symptoms based on BDI were seen in 87 (56.1%) subjects. A total of 71 subjects (45.8%) scored in the severe level of hopelessness (15–20 points). The mean (SD) of SIS was 11.6 (6.1). Twenty one (13.5%) subjects showed high suicidal intent (SIS score > 18). Alcohol and drug use before SB occurred in 14.8% and 9.1% of the subjects, respectively. Means (SDs) of OAS-M 1, 2, 3 and OAS-M Item 7b were 5.9 (7.0), 3.5 (2.8), 8.3 (2.9) and 1.8 (1.3), respectively. The value of OAS-M Item 7b was around “mild, 2”. OAS-M1 and OAS-M 2 scores negatively correlated to age, with coefficients of -0.164 (p < 0.05) and -0.255 (p < 0.01), respectively. Further details of the clinical and socio-demographic data were presented in our previous report [19].

### Construction of MSBS

Table 2 presents the factor structure produced from EFA of MSBS along with response rates and ICCs of the items. At this stage of analysis, as shown in the note for Table 2, nine original MSBS items with poor test-retest reliability scores and Item 13 (suicide intent) that was redundant with SIS and OAS-M 3, were excluded from EFA.

**Table 2:** Factor structure of the original Motivations for Suicidal Behaviors Scale (MSBS).

	IT	IC	SR	SS	Freq. (%)	ICC
**To make others understand you. (Item 15)**	**.807**	.479	.001	.444	54 (34.8)	0.811
**To get attention. (Item16)**	**.784**	.442	.027	.360	34 (21.9)	0.582
**To find out whether someone really loved you or not. (Item 27)**	**.713**	.327	.221	.306	26 (17.4)	0.571
**To seek help from someone. (Item17)**	**.672**	.301	-.076	.221	37 (23.9)	0.616
**To see what others will do in response to the (suicidal) behavior. (Item 26)**	**.613**	.286	.191	.202	18 (11.6)	0.832
**To make people understand how desperate you were feeling. (Item 19)**	.544	.540	.257	.299	56 (36.1)	0.640
**To make people sorry for the way they have treated you, or to frighten or get your own back on someone. (Item 23)**	.383	**.895**	.097	.086	31 (20.0)	0.801
**To make others compensate for what they did to you. (Item 22)**	.410	**.885**	-.062	.124	22 (14.2)	0.769
**To influence a particular person or get them to change their mind. (Item 24)**	.447	**.707**	.180	.215	35 (22.6)	0.849
**To punish yourself. (Item 08)**	.163	.080	**.874**	.348	60 (38.7)	0.799
**To take responsibility for what you did. (Item 09)**	.036	.092	**.786**	.309	57 (36.8)	0.788
**To make things easier for others. (Item 25)**	.218	.077	**.628**	.169	34 (21.9)	0.650
**To have a feeling of living, and to assure yourself that you are living. (Item 11)**	.477	.077	.200	**.738**	18 (11.6)	0.556
**To retrieve a sense of being yourself. (Item 14)**	.254	.194	.181	**.666**	16 (10.3)	0.686
**To feel something, even if it was pain. (Item 10)**	.310	.020	.420	**.627**	30 (19.4)	0.634
**To get control of a situation. (Item 20)**	.100	.029	.209	**.565**	19 (12.2)	0.687
**To show how much you loved someone. (Item 18)**	.454	.194	.190	.181	24 (15.5)	0.640

The factor structure derived from exploratory factor analysis based on maximal likelihood-extraction with Promax rotation is shown in this Table. Factor loadings greater than 0.5, are indicated in bold.

Freq. (%): Frequency (percentage) of the response “clearly present”.

ICC: Intraclass Correlation Coefficient, IT: Interpersonal-testing, IC: Interpersonal-change, SR: Self-renunciation, SS: Self-sustenance.

Items (Freq. (%), ICC) that were excluded from this study with insufficient reliability, were "To stop bad feelings (Item 01) (44 (28.4), 0.395)", "To relieve numb or empty feelings (Item 02) (48 (31.0), 0.295)", "To feel relaxed (Item 03) (21 (13.5), 0.176)", "To get relief from a terrible state of mind (Item 04), (50 (32.3), 0.048)", "You could not keep yourself in the terrible situation (Item 05) (107 (69.0), 0.469)", "The situation was so unbearable that you had to do something and didn't know what else to do (Item 06) (105 (67.7), 0.135)", "To escape for a while from an impossible situation (Item 07) (112 (72.3), 0.272)", "To recover the power of self-control (Item 12) (12 (0.08), 0.177)" and "To get other people to act differently or change (Item 21) (15 (0.10), 0.241)".

“To die (Item 13) (95 (61.3), 0.587)” was also excluded because this item was redundant in other SB-related scales.

Items 4, 6, 7, 17, 18, 19, 23, 24, 25 and 27 were common to the items of Bancroft et al. [4]. Items 1, 2, 3, 8, 9, 10, 16, 20 and 21 were common to the items of Nock and Prinstein [9]. (underlined items were used in the final model.)

The EFA identified four factors with an eigenvalue greater than one, and scree-plot examination also endorsed this factor solution. Approximately 64 percent of the total variance was explained by these four factors.

Factors were labeled as Interpersonal-testing (IT), Interpersonal-change (IC), Self-renunciation (SR) and Self-sustenance (SS). Moderate pairwise correlations were found between IT, and IC and SS, and between SR and SS (0.480, 0.447 and 0.332, respectively). Other pair-wise correlations were weak and non-significant. At this point in analysis, Items 19 and 18 were removed from the next model because of their respective large cross-loading on IT and IC, and inadequate loading on any factor.

**Fig 1:**
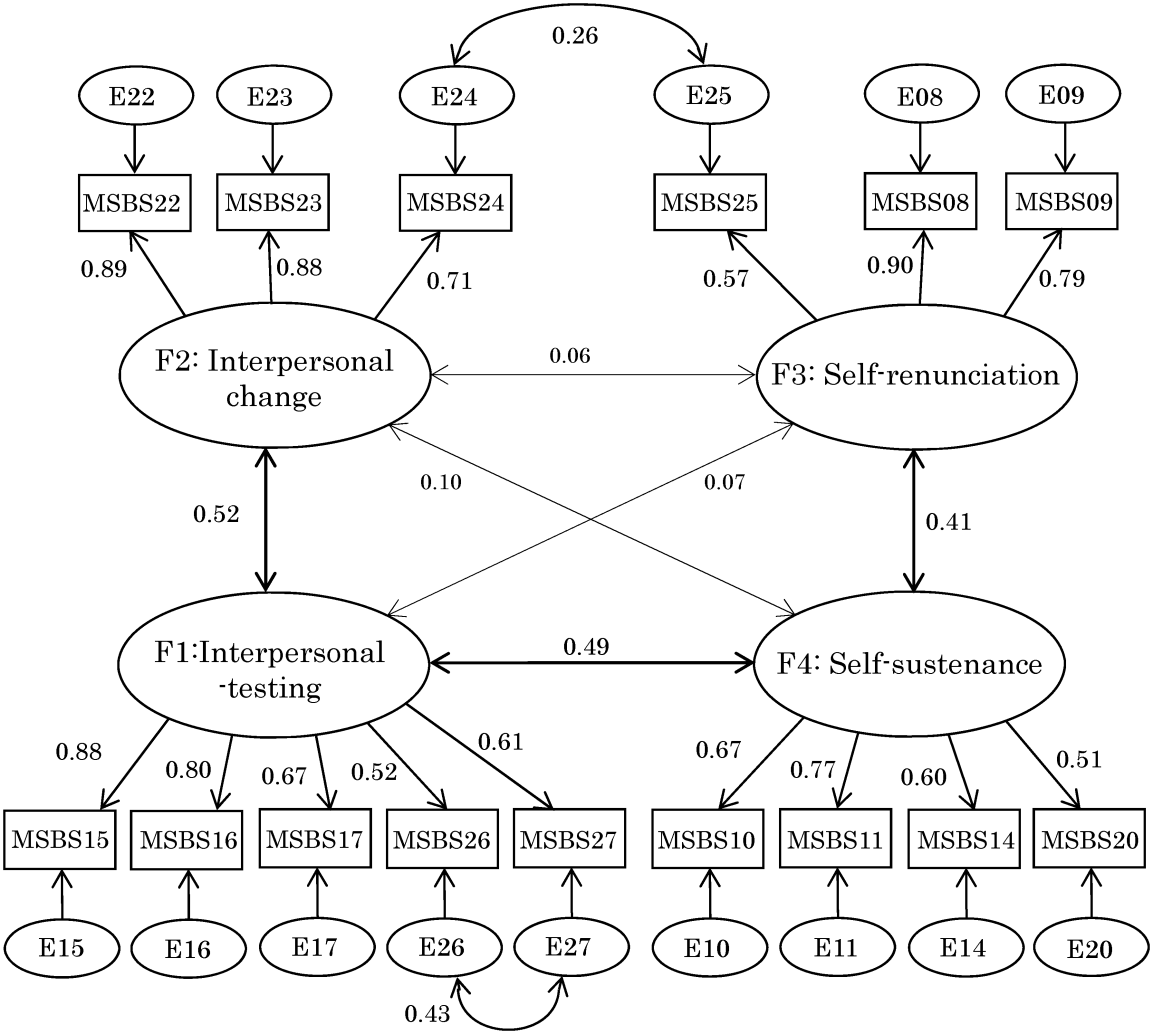
Confirmatory factor analysis (CFA) model of Motivations for Suicidal Behavior Scale (MSBS). Standardized Maximum likelihood (ML)-based parameter estimates are shown in this Fig. Non-significant inter-factor covariates (paths) are indicated by a thin line. MSBS Items were “To punish yourself (Item 08)”, “To take responsibility for what you did (Item 09)”, “To feel something, even if it was pain (Item 10)”, “To have a feeling of living, and to assure yourself that you are living (Item 11)”, “To retrieve a sense of being yourself (Item 14)”, “To make the others understand you (Item 15)”, “To seek help from someone (Item 17)”, “To get attention (Item 16)”, “To show how much you loved someone (Item 18)”, “To make people understand how desperate you were feeling (Item 19)”, “To get control of a situation (Item 20)”, “To make others compensate for what they did to you (Item 22)”, “To make people sorry for the way they have treated you, or to frighten or get your own back on someone (Item 23)”, “To influence a particular person or get them to change their mind (Item 24)”, “To make things easier for others (Item 25)”, “To see what others will do in response to the (suicidal) behavior (Item 26)” and “To find out whether someone really loved you or not (Item 27)”.

The final CFA model is shown in Fig 1. All estimates excluding three inter-factor covariates (IT—SR, IC—SR and IC—SS) were statistically significant (p < 0.01). Two inter-error covariances (E26—E27 and E24—E25) were set, after which significant reductions in the χ2 statistic ensued (26.95 (df = 1, p < 0.001) and 8.52 (df = 1, p = 0.002), respectively).

CFI and RMSEA in the final model were 0.952, and 0.060, respectively, which indicated a permissible or favorable level of goodness of fit for the model. However, the model’s χ2 statistic did not reach a non-significant level of probability (χ2 = 128.16, df = 82, p = 0.001).

Cronbach’s alpha coefficients of the subscales; IT, IC, SR and SS were 0.868, 0.704, 0.801 and 0.804, respectively, which indicated a permissible or favorable internal consistency according to Nunnally and Bernstein [30]. Correlations of the four MSBS subscales with the respective factor scores calculated using the factor score weight matrix were adequately high (0.958, 0.982, 0.968 and 0.959, respectively). Means (SDs) of the subscales were 3.2 (3.1), 1.6 (2.1), 2.5 (2.2) and 1.6 (2.1), respectively.

### Convergent validity and clinical relevance of MSBS

Table 3 shows the results of correlation analyses between MSBS subscales and SB-related clinical characteristics. Interpersonal subscales (IT and IC) correlated negatively with suicidality (SIS and OAS-M 3), and positively with overt aggression (OAS-M 1 and 2). We detected correlations of SR with depressive symptomatology (DBI) and SIS, while SS showed no correlation with any variables of suicidality, overt aggression or depression.

**Table 3:** Correlation analyses of MSBS subscale scores and SB-related clinical characteristics.

	IC	SR	SS	BDI	BHS	SIS	OAS-M1	OAS-M2	OAS-M 3
**Interpersonal-testing (IT)**	0.457**	0.114	0.332**	-0.147	-0.137	-0.365**	0.256**	0.272**	-0.350**
**Interpersonal-change (IC)**	1.000	0.112	0.113	0.007	-0.060	-0.157	0.191*	0.218**	-0.227**
**Self-renunciation (SR)**		1.000	0.331**	0.357**	0.103	0.258**	-0.015	0.024	0.119
**Self-sustenance (SS)**			1.000	0.102	-0.135	0.049	0.032	0.035	0.020
**BDI Total score**				1.000	0.484**	0.316**	-0.081	-0.038	0.279**
**BHS Total score**					1.000	0.174*	-0.164*	-0.093	0.122
**SIS score**						1.000	-0.258**	-0.255**	0.616**
**OAS-M 1**							1.000	0.792**	-0.090
**OAS-M 2**								1.000	-0.080
**OAS-M 3**									1.000

BDI: Beck Depression Inventory-II, BHS: Beck Hopelessness Scale, SIS: Suicide Intent Scales, OAS-M: Overt Aggression Scale-Modified. OAS-M 1: Aggressive behavior, OAS-M 2: Irritability, OAS-M 3: Suicidal tendencies

* p < 0.05,

** p <0.01 (two-tailed)

Tables 4, 5 and 6 present MANCOVAs of MSBS subscales with psychiatric disorder groups, interpersonal RLEs and SB methods. Among the psychiatric disorder groups, BPD showed significant associations with IC and SS (Table 4). We detected significant association of RLE discord or conflict with IT, and RLE separation or death with SR (Table 5). When self-strangulation was set as a reference SB method, self-cutting was associated with IT, and Overdosing, with SS (Table 6).

**Table 4:** Multivariate Analysis of Covariance (MANCOVA) regression of MSBS in relation to psychiatric and personality disorders (groups).

Motivations	Psychiatric disorders	B	p	95% CI	Partial Eta Squared
**Interpersonal-testing (IT)**	Affective disorders	-0.530	0.324	-1.588 - 0.528	0.007
	Anxiety disorders	1.036	0.069	-0.081 - 2.153	0.022
	BPD	1.144	0.059	-0.046 - 2.333	0.024
**Interpersonal-change** **(IC)**	Affective disorders	-0.589	0.103	-1.299 - 0.121	0.018
	Anxiety disorders	0.679	0.076	-0.071 - 1.428	0.021
	BPD	0.922	0.024	0.124 - 1.721	0.034
**Self-renunciation (SR)**	Affective disorders	0.186	0.630	-0.575 - 0.947	0.002
	Anxiety disorders	0.768	0.061	-0.036 - 1.571	0.023
	BPD	0.621	0.153	-0.234 - 1.477	0.014
**Self-sustenance (SS)**	Affective disorders	-0.439	0.234	1.165 - -0.287	0.010
	Anxiety disorders	0.548	0.160	-0.218 - 1.314	0.013
	BPD	0.872	0.036	0.056 - 1.688	0.029

Regression coefficients of covariates (gender and age) were not statistically significant.

MSBS: Motivations for Suicidal Behaviors Scale, 95% CI: 95% Confidence Interval, BPD: Borderline Personality Disorder

**Table 5:** Multivariate Analysis of Covariance (MANCOVA) regression of MSBS in relation to interpersonal recent life events.

Motivations	Interpersonal RLE	B	p	95% CI	Partial Eta Squared
**Interpersonal-testing (IT)**	Discord or conflict	1.239	0.024	0.169–2.310	0.034
	Separation or death	-0.696	0.210	-1.787–0.395	0.010
**Interpersonal-change (IC)**	Discord or conflict	0.721	0.052	-0.007–1.449	0.025
	Separation or death	-0.365	0.333	-1.107–0.377	0.006
**Self-renunciation (SR)**	Discord or conflict	-0.304	0.437	-1.075–0.467	0.004
	Separation or death	0.920	0.022	0.134–1.706	0.034
**Self-sustenance (SS)**	Discord or conflict	-0.165	0.660	-0.904–0.575	0.001
	Separation or death	0.674	0.079	-0.079–1.428	0.020

Regression coefficients of covariates (gender and age) showed no statistical significance.

MSBS: Motivations for Suicidal Behaviors Scale, 95% CI: 95% Confidence Interval, RLE: Recent Life Event

**Table 6:** Multivariate Analysis of Covariance (MANCOVA) regression of MSBS scores in relation to SB methods.

Motivations	SB methods ^a^	B	P	95% CI	Partial Eta Squared
**Interpersonal-testing** **(IT)**	Overdosing	1.471	0.080	-0.180–3.121	0.020
	Self-cutting	1.838	0.022	0.263–3.412	0.034
	Others	1.647	0.057	-0.052–3.344	0.024
**Interpersonal-change (IC)**	Overdosing	0.544	0.341	-0.580–1.668	0.006
	Self-cutting	0.895	0.101	-0.178–1.967	0.018
	Others	0.336	0.567	-0.820–1.492	0.002
**Self-renunciation (SR)**	Overdosing	-0.538	0.378	-1.741–0.664	0.005
	Self-cutting	-0.476	0.414	-1.622–0.671	0.004
	Others	-0.089	0.887	-1.325–1.147	0.000
**Self-sustenance (SS)**	Overdosing	1.479	0.010	0.354–2.604	0.043
	Self-cutting	1.038	0.058	-0.035–2.111	0.024
	Other methods ^b^	1.075	0.068	-0.081–2.232	0.022

Regression coefficients of covariates (gender and age) were not statistically significant.

MSBS: Motivations for Suicidal Behaviors Scale, 95% CI: 95% Confidence Interval, SB: Suicidal behavior

^a^ The reference category is self-strangulation in this analysis.

^b^ Predominant methods were ‘jumping from a height’ and ‘attempting death in traffic’.

The main effects of BPD and separation or death of RLE on the combined dependent variables were statistically significant (F_4, 147_ = 2.449, p = 0.049; Wilks' Lambda = 0.937 and F_4, 147_ = 2.760, p = 0.030; Wilks' Lambda = 0.930, respectively). However, the variables of RLE discord or conflict and SB method did not reach significant levels (F_4, 147_ = 2.155, p = 0.077; Wilks' Lambda = 0.945 and F_12, 386.6_ = 1.368, p = 0.179; Wilks' Lambda = 0.896, respectively.)

These results highlighted that the final CFA model constructed from the MSBS showed permissible model-fit indices and that the subscales demonstrated sound psychometric properties. The correlation analyses of MSBS subscales also confirmed their convergent validity, and accordingly ensured their clinical relevance.

## Discussion

### Features of motivations for SB

This study presented a construct for MSBS with the aim of measuring features of motivations associated with SB in psychiatric patients, using a factor analytical model. The relevant features were Interpersonal-testing (IT), Interpersonal-change (IC), Self-renunciation (SR) and Self-sustenance (SS). They can be arranged by the distinctions between interpersonal versus intra-personal directedness (IT and IC vs. SR and SS) and the levels of assumed influence (minimal vs. greater) by SB (IT and SS vs. IC and SR).

The features represented on MSBS subscales are characterized by their link to suicidality. SR was moderately related to suicidality, which is conceivable since both share an aspect of self-negation. In addition, the inverse relationship between the interpersonally directed motivations (IT and IC) and suicidality is plausible if they are regarded as opposite positions of directedness.

Negative emotions, such as anger and depressive feelings represented by overt aggression and depression in this study, are also distinguishing features. SR was related positively to depression, while IT and IC were related positively to aggression. Based on Lazarus’s assertion that motivations are formed in relation to emotions [13], anger and depressive feelings were considered integral to the SB motivating process. The correlations between motivation features and emotions found in this study parallel those found in the psychology of emotions [31]: depressive feelings principally concern self, and are linked to self-depreciation; aggression (anger) is mainly directed at external objects and other individuals. In contrast, SS motivations that aimed at soothing inner distress appeared to be independent of suicidality or a particular emotional state.

The degree of assumed influence by SB can also be extracted from the relationship to prevailing emotions. Motivations with a minimal influence (IT and SS) that seek to confirm or restore previous interpersonal and intra-personal conditions, are used to neutralize or counteract anger and a lacking sense of self (numbness or emptiness), intensified in the period preceding SB. In contrast, motivations with a greater influence (SR and IC) are thought to be driven by excessive negative emotions, like anger and guilt, which are released to attain relief in SB. When analyzed together, this distinction can be defined as that of emotion-counteracting motivations versus emotion-driven motivations.

As discussed previously, the nature of motivations vary depending on the type of SB and the sampled population. Although variability in inquiry items, studied samples and analytic methods makes direct comparisons difficult, we are still able to examine similarities and differences among the factor compositions of other studies.

Table 7 presents the results of factor analytic studies on the motivations of collated clinical populations. Extracted factors are first classified by interpersonal versus intra-personal directedness and then by the level of assumed influence (change) by SB (minimal vs. greater) or the relation to emotion (emotion-counteracting vs. emotion-driven).

**Table 7:** The factor analytic studies of the motivations for SBs of clinical populations

Authors, Scale name, Number of items and factors, Studied population (If presented, frequent SB methods)	Interpersonal factors (labels)		Intrapersonal factors (labels)	
	Minimal change Emotion-counteracting	Greater change Emotion-driven	Minimal change Emotion-counteracting	Greater change Emotion-driven
Holden & Kerr (1998), “Reasons for Attempting Suicide”, 14 items, 2 factors, 173 patients presenting with suicide attempts or ideations	Extrapunitive / manipulative reasons (6 items)		None	Internal perturbations (8 items)
Osuch et al. (1999), SIMS, 36 items, 5 factors, 99 psychiatric patients admitted with SIB (Self-cutting, hitting, burning and substance abuse)	Influencing others (5 items) Magical control (7 items)^a^		Affect modulation (9 items)^b^, Desolation-easing (4 items), Self-stimulation (4 items)^c^	Punitive duality (6 items)^d^
Hjelmeland et al. (2002), MPQ, 14 items, 4 factors, 1646 patients presenting with parasuicide (Mostly, overdosing)	Care seeking (4 items)	Influencing others (3 items)	Temporary escape (3 items)^e^	Final exit (4 items)
McAuliffe et al. (2007), MPQ, 14 items, 4 factors, 146 patients presenting with DSH (Mostly, overdosing)	Appeal (4 items)	Revenge (2 items)	Interruption (4 items)^e^^,^ ^f^	Escape (4 items)
Nixon et al. (2015), OSI, 22 items, 4 factors, 94 adolescent inpatients with NSSI	Social influence (7 items)	External emotion regulation (3 items ^g^)	Internal emotion regulation (7 items)^b^ Sensation seeking (3 items)^c^	
Klonsky et al. (2015), ISAS, 39 items (13 functions), 2 factors, 946 mostly adolescent patients with NSSI (Mostly, self-cutting and scratching)	Interpersonal factor (3 purely interpersonal functions)		(5 functions)^h^	Intrapersonal factor (5 functions)
This study, MSBS, 15 items, 4 factors, 155 psychiatric patients admitted with SB (Overdosing, self-cutting and self-strangulation)	Interpersonal-testing (IT) (5 items)	Interpersonal-change (IC) (3 items)	Self-sustenance (SR) (3 items)	Self-renunciation (SS) (4 items)

MPQ: Motives for Parasuicide Questionnaire, SIMS: Self-Injury Motivation Scale, OSI: Ottawa Self-Injury Inventory, ISAS: Inventory of Statements About Self-injury, MSBS: Motivations for Suicidal Behavior Scale.

SB: Suicidal Behavior, SIB: Self-Injurious Behavior, DSH: Deliberate Self-Harm, NSSI: Non-Suicidal Self-Injury.

The classification applied in this table is from the 4-factor model described in this study. Two distinctions: interpersonal vs. intra-personal directedness, and the level of assumed change (influence) by suicidal behavior (minimal vs. greater) or the relation to emotion (emotion-counteracting vs. emotion-driven) were used.

The study of Nock & Prinstein [9] is not included in this table since it did not undertake any novel explorative analysis, but instead statistically confirmed the authors’ theoretical model.

^a^ 3 items in this factor that pertain to the intrapersonal motivations are included here.

^b^ The item “To punish yourself” in these factors that pertains to the intra-personal and greater change-assumed (emotion-driven) motivations is included here.

^c^ 2 items of generating especially positive feelings: “sense of exhilaration” and experiencing a “high” are included in these factors.

^d^ The item “To remind yourself that you are alive” in this factor that pertains to the intra-personal and minimal change-assumed (emotion-counteracting) motivations is included here.

^e^ The item “I do not know why I did it” in this factor cannot be properly classified in this table.

^f^ The item “To let others know how desperate you are” in this factor that pertains to the interpersonal and greater change-assumed (emotion-driven) motivations is included here.

^g^ This factor includes 3 items of releasing strong emotions. Since it is assumed that they exert a strong influence on others, the factor is placed in this category of interpersonal and greater change-assumed (emotion-driven) motivations.

^h^ These functions are Autonomy, Marking distress, Self-care, Sensation-seeking and Toughness.

This table indicates that our classification is largely applicable to previous studies, whereas several factors included a few incongruent items as shown in the notes. This classification is especially well-matched to the factor composition studies of Hjelmeland et al. [6] and McAuliffe et al. [7], both of which mainly dealt with patients presenting with overdosing. However, there are considerable discrepancies between the classification and the factors of scales used for assessing NSSI or SIB that were mostly self-cutting [10, 11]. A possible explanation could be that the studies on NSSI or SIB did not deal with a sufficient number of items related to intense inner distress, such as feelings of despair and guilt, most of which would have been included in the intra-personal and greater influence-assumed (emotion-driven) (SR) motivations. Additionally, these studies contain items that record the presence of profitable functions of SB, such as generating positive feelings [10–12] (Table 7 notes). It is most likely that these differences result from a bias in item-selection to capture the features of studied sample in each study. In this study, when constructing our MSBS, the item set was shaped to match the subjects with high suicidality by omitting items that reflected the profitable aspects of SB. In contrast with those views that stress differences in the factors, Bryan et al. [32] reported that the theoretical model for SIB proposed by Nock and Prinstein [9] was applicable for adult suicide-attempters. The question as to whether suicide attempts, and NSSI or SIB have a common factor composition remains open for future investigations.

### Clinical relevance of motivations

The analyses of correlations between features of suicidality, and IT, IC and SR revealed defined association patterns. These patterns were almost identical to those found by Hjelmeland et al. [6] and McAuliffe et al. [7]. Here, suicidality was negatively associated with interpersonal features, and positively with intra-personal and emotion-driven features. This supports data from Holden et al. [5], who showed positive association of suicidality with intra-personal and emotion-driven motivation features. The other studies listed in Table 7 did not examine association between their factors with suicidality.

In line with the negative association of aggression with SR-motivations and suicidality found in this study, Brown et al. [15] reported associations in the same direction in suicidal BPD patients. In contrast, Boerger et al. [14] indicated a positive association between suicidal intent and aggression among suicidal adolescents. This discrepancy can be explained by crucial differences in their study settings. The study of Boerger et al. [14] was based on inception interviews in an emergency clinic where articulating a desire to die was very close to expressing aggression for the adolescents, whereas the adult patients in the two former studies were in an inpatient setting with more time for calmer thoughts before assessments.

Of the psychiatric diagnoses, only BPD showed significant correlation with the motivations of IC and SS. Correlation with the former motivation is most likely due to the BPD pathology that impacts greatly on the interpersonal relationships of patients. The latter correlation with SS motivation is also consistent with characteristics of BPD patients who generally suffer from fragile self-feeling, and periodically make strenuous efforts to restore self-feeling [16]. In patients with BPD, SB led by these motivations is seen as a means of correcting interpersonal relations and inner disequilibrium. Likewise, Sadeh et al. [18] indicated that BPD patients’ interpersonal and intra-personal motivations corresponded to interpersonal dysfunction and a disturbed sense of self, respectively. These data indicate that motivations are formulated to alleviate the persistent distress caused by BPD pathology.

Contrary to our earlier assumption, depressive disorders showed no significant relationship with any motivations. One reason could be that any effect may have been reduced to non-significant levels by BPD because of its strong association with depressive disorders.

Associations between IT motivations, and RLE discord or conflict and between SR, and RLE separation or death are considered to indicate that these motivations are created as reactions to interpersonal life events. An explanation for this would be that SB motivated by SR is designed to reduce the intensity of emotions aroused by the loss of an important person through releasing them, and that SB expressed via IT motivations are to counteract or offset emotions aroused by interpersonal difficulties.

We found associations between different motivations and SB methods, indicating that the motivations played a role in determining the method. Overdose-taking and self-cutting, when compared to self-strangulation, were related to SS and IT motivations, respectively. These results can be understood in terms of the intrinsic property of SB methods. Overdose-taking, which was mostly that of prescribed psychotropics, generally induces sedation. It would be selected for soothing inner distress with SS motivations. On the other hand, self-cutting could potentially leave scars on the skin as an expression on the body surface, possibly explaining why it is selected with IT motivations, such as seeking attention or care. Rodham et al. [33] compared the motivations of self-poisoners and self-cutters in a community adolescent sample, and found that the former had stronger suicide intent and a motivation to escape from the problem, while the latter expressed aggression more frequently. Thus, it is recognized that the SB method was selected in a manner congruent to the motivation.

The links between motivation and various characteristics indicate that motivations are formed to achieve a new interpersonal and intra-personal equilibrium in the face of aversive conditions leading to conducting SB.

Combined data suggests that different approaches are promising for preventing SB based on the distinctive four types of motivation. Implementing more sophisticated coping strategies would be useful for IT and SS-motivated SB, since they are thought to counteract aversive interpersonal and intra-personal conditions. In cases of IC and SR-motivated SB that are instigated by negative emotions, these emotions, such as anger and self-depreciation are to be addressed first. In the cases of SB where interpersonal motivations (IT and IC) play a role, strategies directed at improving interpersonal relationships can offer some protection against SB. In patients who employ RLEs in their motivation (especially IT and SR), exploring distress aroused in RLE, can lead to new coping strategies that may result in reducing SB.

### Limitations

There are several limitations to this study in keeping with work of this nature. Firstly, the size of sample used may be limiting when attempting to examine a wide range of motivation features. The presented model needs extension as well as replication by future studies utilizing larger samples. Secondly, since this study was based on self-reporting, it is prone to recall biases and thus inherent data distortion. In this respect, the effects of substance use and dissociation, not uncommon features of the studied sample [19], may have increased recall bias and consequently affected our results. Thirdly, our results were derived from a cross-sectional study, and would not be able to determine causal relationships, with some exceptions of situationally or temporarily assumable causal relations. Therefore, care needs to be taken when interpreting this data in a simplistic cause-effect context. Additionally, considering the complexity of the interactions between motivations and other SB-related factors in individual cases, it is evident that the hypothesis linking these interrelationships needs further scrutiny. Particularly, since the motivations for behavior referred to in this study were subjectively derived and not actual causal factors. Lastly, the major limitation we encountered in conducting this study was that many items were excluded from model construction due to insufficient reliability and low response rate, resulting in a narrowing of the range of motivations available for analysis. One possible explanation is that items derived from the study of SIB with low suicidality, particularly those related to SB-profiting aspects, were not well-suited to the subjects of this study, from which relatively low response rate and reliability ensued. There is also another possibility that our subjects’ perception of motivations may have changed because of ongoing psychiatric treatment conducted actively during the test-retest interval of this study.

### Conclusions

In this study, we constructed MSBS by using the four-factor model, with permissible model-fit indices and sound psychometric properties. The results of this study support the hypothesis that motivation for SB is integral to the development of SB, and that this is in turn linked to related factors such as emotional state, psychiatric disorders, interpersonal life events and SB method selection. The four features of motivation extracted from this study were classified by interpersonal versus intra-personal directedness, and the level of assumed influence, namely a minimum or greater level and the relationship to emotion, in this case, emotion-counteracting versus emotion-driven. This model has great potential to improve our understanding of the complicated formative processes of SB. In time, this will hopefully lead to more fine-tuned treatment and research approaches for SB patients.

## Supporting information

S1 Dataset MSBS. The data set for analyses except that for the reliability study. The first line indicates variable labels.
(XLSX)

S2 Dataset MSBS reliability study. The data set for the MSBS reliability study. The first line indicates variable labels.
(XLSX)
